# Prognostic significance of collagen content in solitary fibrous tumors of the central nervous system

**DOI:** 10.3389/fonc.2024.1450813

**Published:** 2024-11-12

**Authors:** Xiaoling Li, Hua Zhang, Chengcong Hu, Liwen Hu, Huibin Guo, Hongbao Chen, Guoping Li, Qian Huang, Shuie Jiang, Sheng Zhang, Zhen Xing, Xingfu Wang

**Affiliations:** ^1^ Department of Pathology, The Second Hospital of Longyan, Longyan, Fujian, China; ^2^ Department of Pathology, The First Affiliated Hospital of Fujian Medical University, Fuzhou, Fujian, China; ^3^ Department of Pathology, First Affiliated Hospital, National Regional Medical Center, Fujian Medical University, Fuzhou, Fujian, China; ^4^ Department of Radiology, The First Affiliated Hospital of Fujian Medical University, Fuzhou, Fujian, China; ^5^ Department of Radiology, First Affiliated Hospital, National Regional Medical Center, Fujian Medical University, Fuzhou, Fujian, China; ^6^ Department of Pathology, Jianning General Hospital, Sanming, Fujian, China

**Keywords:** solitary fibrous tumors, collagen content, pathology, prognostic, radiomics

## Abstract

**Purpose:**

We aimed to explore the prognostic significance of collagen content in solitary fibrous tumors (SFTs) of the central nervous system (CNS) and preliminarily investigate its relationship with magnetic resonance imaging (MRI) features of SFTs.

**Methods:**

Collagen content was identified using Masson’s trichrome staining, and quantitatively assessed. Radiomic methods were applied to extract quantitative MRI features of SFTs, which were then analyzed in relation to collagen content.

**Results:**

The collagen content in CNS SFTs was categorized into high- and low-content groups, with a cutoff value of 6%. Survival analysis indicated a positive correlation between collagen content and overall survival (OS). In multivariate Cox regression analysis, incorporating factors such as mitosis, necrosis, Ki67, and collagen content and other indicators, collagen content emerged as an independent prognostic factor. Collagen content demonstrated a negative correlation with tumor histological phenotype, Ki67, WHO grade, mitosis, necrosis, and brain invasion. Additionally, the signal intensity of SFTs on T2-weighted imaging (T2WI) decreased with increasing collagen content. Radiomics analysis identified 1,702 features from each patient’s region of interest, with 12 features showing significant differences between the high and low collagen content groups. Among the quantitative parameters and radiomic models, the combined T1- and T2WI models exhibited the highest diagnostic performance.

**Conclusion:**

These findings suggest that collagen content is an independent prognostic risk factor for OS. Furthermore, combined radiomic models based on T1-and T2WI sequences may offer a more comprehensive, objective, and accurate assessment of collagen content in CNS SFTs.

## Introduction

Solitary fibrous tumors (SFTs) of the central nervous system (CNS) account for less than 1% of all CNS primary tumors. They predominantly occur in individuals aged 40–60 years and show no clear sex preference (1, 2). In the 2021 World Health Organization (WHO) classification of CNS tumors, the term “hemangiopericytoma” (HPC) was eliminated and aligned with soft tissue nomenclature, merging both entities into SFT, with a simplified histological grading system based on mitotic count and necrosis categorized into three grades (3). However, compared with SFTs outside the CNS, those in the CNS exhibit higher rates of local recurrence and distant metastasis rates (4). Furthermore, the prognostic risk models proposed by Demicco et al. in 2015 and 2017 for soft-tissue SFTs (5, 6) are not applicable to CNS SFTs (3), as variables such as age and tumor size, which are included in these models, are not associated with patient prognosis (1, 7). In recent years, several studies have proposed risk stratification models for SFTs (8, 9), including recommendations to incorporate molecular testing and the Ki67 index (10, 11). These studies primarily focus on prognostic factors related to tumor recurrence and metastasis. Additional research is necessary to improve the ability to predict the prognosis of patients with CNS SFT, especially in relation to overall survival (OS).

Surgical resection is the primary treatment for CNS SFTs (12). Preoperative prediction of tumor grade through imaging studies plays a critical role in treatment planning and prognostic assessment. Magnetic resonance imaging (MRI) is a vital preoperative imaging tool for CNS tumors, offering high soft-tissue resolution and the ability to non-invasively reflect tumor phenotypic features. The integration of computer science, mathematics, statistics, and big data imaging has led to the development of radiomics, which has brought artificial intelligence into the imaging field. Radiomics is centered on the essence of structural heterogeneity analysis, allowing for the non-invasive extraction of extensive feature data from traditional imaging, that is imperceptible to the naked eye (13). This data is subjected to quantitative analysis, providing a more objective basis for clinical decision-making (14) compared to traditional imaging methods. Radiomics is widely used in disease diagnosis, efficacy monitoring (15), prognostic evaluation (16), gene prediction (17), and postoperative complication assessment (18). Numerous studies have focused on CNS SFT radiomics, particularly on differentiating meningiomas (19, 20). However, classification and prognostication of SFTs remain largely reliant on conventional imaging data (21). A recent study (22) demonstrated that a comprehensive model incorporating clinical data, radiomics, and deep learning enhanced the preoperative histological grading of SFTs. Nevertheless, since this study applied the older WHO grading criteria, and the grading standard has since undergone significant changes, further research is needed to evaluate radiomic-based grading and prognosis of SFTs under the updated standards.

CNS SFTs typically present as isointense on T1-weighted imaging (T1WI) and heterogeneous on T2-weighted imaging (T2WI) (23, 24). The heterogeneity observed in T2WI signals reflects the tumor’s histological characteristics, closely linked to the content and distribution of tissue components, including tumor cells, collagen, and blood vessels. Regions rich in tumor cells show slightly higher signals, whereas collagen-rich areas produce lower signals. As. collagen content increases, the signal intensity of SFTs on T2WI decreases.

Collagen fibers are the primary constituents of the extracellular matrix, and many soft tissue tumors, including SFTs, contain collagen fibers. The collagen content in SFTs varies significantly, displaying diverse morphologies. To date, research on collagen within the brain has mainly focused on its role as an important biomaterial in 3D cell culture models of brain tumors and brain cell growth, emphasizing that its rheological properties and interactions with cells are crucial for cell behavior and tissue engineering (25, 26). However, no studies have specifically focused on the collagen content in SFT. Given the characteristic low-signal appearance of collagen on T2WI and their significance as a major component of SFTs, it is important to investigate whether collagen content correlates with the WHO grading system, as well as the biological behavior and prognosis of SFTs. It is also plausible that collagen content may influence treatment approaches or drug dosages for patients, although this requires further research.

In this study, we used image analysis software and computer-aided objective quantitative analysis to objectively assess the collagen content of SFTs. We examined whether collagen content was associated with various clinicopathological indicators, whether it could serve as an independent prognostic marker for SFTs, and whether collagen content and MRI features were correlated using quantitative and radiomic methods. Our goal was to identify the diagnostic marker for grading and to elucidate the pathological basis of MRI findings.

## Materials and methods

### Study population

A retrospective consecutive collection of 82 archived cases diagnosed as CNS SFTs was obtained from the Pathology Department of the First Affiliated Hospital of Fujian Medical University between March 2006 and June 2021. The inclusion criteria were as follows: (1) pathologically confirmed diagnosis of SFTs based on surgical tissue pathology, with positive immunohistochemistry for STAT6; (2) initial surgical treatment without prior therapy; and (3) sufficient tissue for inclusion in tissue microarray analysis. The exclusion criteria were as follows: (1) cases that underwent biopsy without surgical resection; (2) perioperative death or death during follow-up due to unrelated causes; and (3) lack of follow-up data.

### Clinical and pathological data

**Figure 1:**
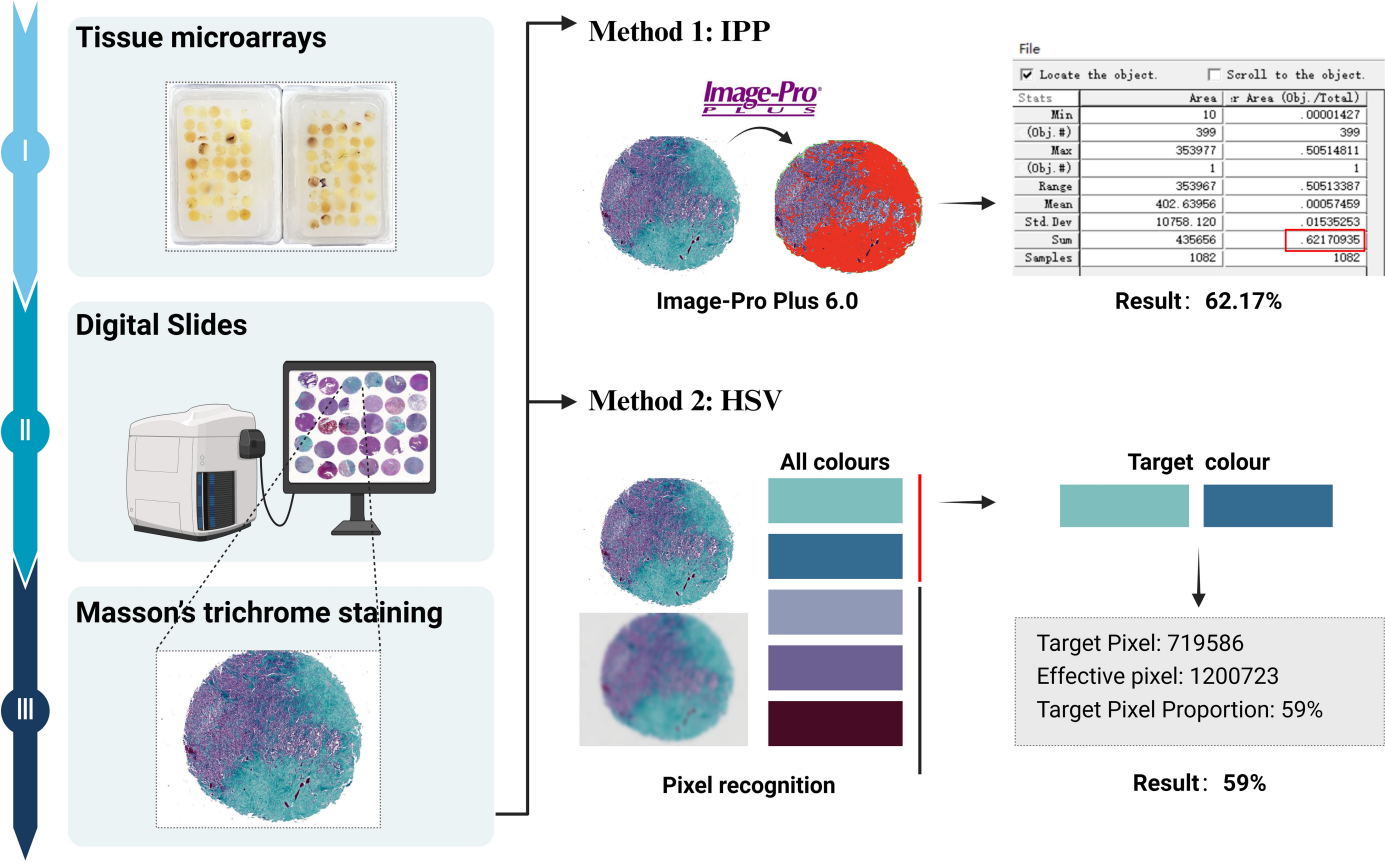
Flow chart of collagen quantification: The tissue microarrays are stained with Masson’s trichrome, and digital pathological sections were obtained. Collagen fibers are stained as green or cyan. Collagen content is quantitatively analyzed using the IPP software, and the ratio of collagen is calculated using the HSV color model algorithm.

Clinical data for all patients were obtained from medical records. Routine hematoxylin and eosin (H&E)-stained slides and immunohistochemical slides were independently reviewed by two experienced pathologists, each with 15 and 10 years of experience, respectively. In cases where grading opinions differed, a consensus was reached through discussion. Follow-up data were tracked and recorded by a third pathologist. Tissue microarrays were prepared for all cases, with representative areas selected for three tissue cores per case, each with a diameter of 0.2 cm at 6×7 positions. Subsequently, H&E and Masson staining were performed, and digital pathology sections were obtained using an automatic digital slide scanner (Image-Pro Plus [IPP]; Media Cybernetics, Rockville, MD). The hue, saturation, and value (HSV) color model algorithm was applied to calculate the ratio of collagen (**Figure 1**).

### Imaging data

A retrospective analysis of 45 patients with SFTs was conducted using pathological data and routine MRI examinations. Patients underwent head MRI scans using magnetic resonance scanners with field strengths of 0.5, 1.5, and 3.0 T. Conventional MRI plain scan sequences included transverse T2WI, transverse T1WI, and sagittal T1WI. According to the 2021 WHO classification standard (3), 23 cases were classified as grade 1, seven as grade 2, and 15 as grade 3 disease. Two neuroradiologists with 9 and 5 years of experience independently performed the image analysis while being blinded to the clinical and pathological data.

### Quantitative assessment

First, on T2WI, the maximum tumor slice was selected, and a region of interest (ROI) was delineated along the tumor’s edge, carefully avoiding areas of calcification, hemorrhage, cystic changes, and necrosis, to obtain the tumor T2 value. Second, a round ROI (30–50 mm^2^) was delineated in the contralateral normal thalamus and centrum semiovale, and the T2 values of the thalamus and centrum semiovale were acquired. The relative T2 value of the tumor was derived using the following formulas: rT2 _thalamic_ = T2 value of the tumor/T2 value of the thalamus, and rT2 _centrum semiovale_ = T2 value of the tumor/T2 value of the centrum semiovale. Similar methods were used to obtain relative T1 values.

### Radiomics analysis

**Figure 2:**
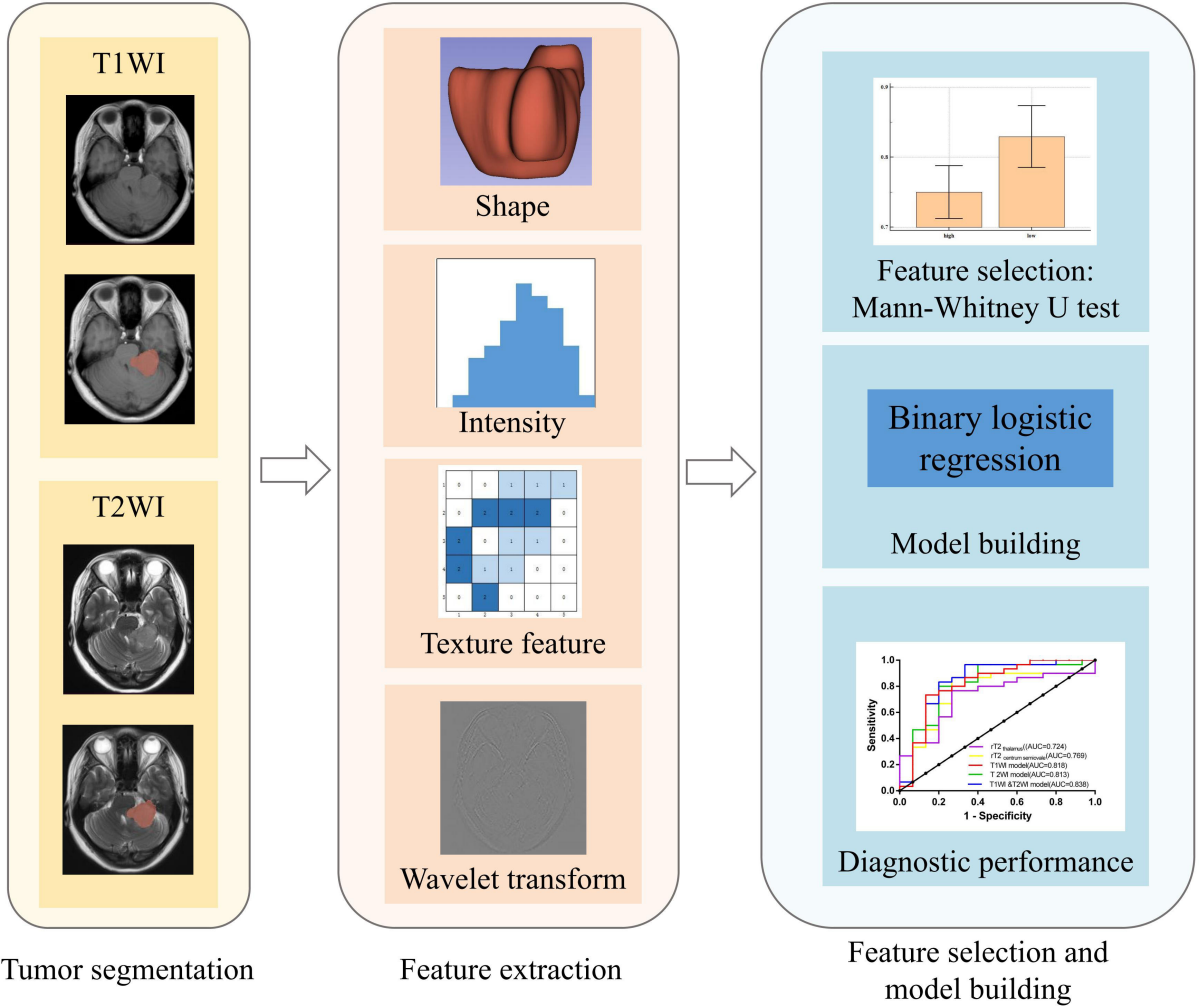
Flow chart of radiomics.

**Figure 2** illustrates the radiomic analysis process. First, SPM12 on Matlab R2016b (MathWorks, Natick, MA) was used for the registration and voxel normalization of T1WI and T2WI. Second, all T1WI and T2WI data were exported to 3D Slicer (version 4.8.0, http://www.slicer.org) software. A physician outlined the tumor layer-by-layer along the edges on T2WI and T1WI, excluding areas of necrosis, cystic changes, and hemorrhage, among others, to obtain the three-dimensional volume of interest (VOI). Third, the T1WI, T2WI, and VOI were imported into an open-source software, FeAture Explore, for quantitative radiomic feature extraction using the Pyradiomics module in Python (3.7.6) (27). A total of 851 features were extracted from each sequence image, consisting of 18 first-order statistics features, 14 shape-based features, 75 texture features, and 744 wavelet features from eight wavelet-transformed images. The details of the extracted features are listed in the **Supplementary Material** (**Supplementary Table 1**). Lastly, we performed a selection using univariate analysis with the Mann-Whitney *U* test. This filtering step filtered out many irrelevant features with the *P*-value threshold set at 0.05. Features with a *P* < 0.05 were identified and selected for further analysis.

### Statistical analyses

Statistical analysis was performed using SPSS 21.0 software. The Kaplan-Meier method was applied for survival analysis and log-rank tests were used to compare survival curves. Prognostic factors of SFTs were analyzed using Cox proportional hazard regression analysis. Spearman’s bivariate analysis was conducted for assessment analysis, and measured data were tested for normality and homogeneity of variance. Data conforming to a normal distribution were represented as x ± s, and an independent sample t-test was performed. Non-normally distributed data were presented as medians ± interquartile ranges and compared using the non-parametric Mann-Whitney U test. Categorical data were compared using the chi-squared test. The intragroup correlation coefficient (ICC) was used to evaluate the consistency of measurements between the two physicians for quantitative data. Binary logistic regression models were constructed to analyze the T1WI, T2WI, and combined T1WI and T2WI radiomic models. Receiver operating characteristic (ROC) curves were used to evaluate the efficacy of the classification model, including the area under the ROC curve (AUC), accuracy, sensitivity, specificity, positive predictive value (PPV), and negative predictive value (NPV). The Delong test was used to assess the differences between the joint models. Statistical significance was set at *P* < 0.05.

## Results

Among the 82 CNS SFT patients, 69 met the inclusion criteria. Four patients who had not received surgical treatment for the first time, one patient without follow-up data, and eight patients with insufficient tissue volume were excluded.

### Clinicopathological features

The ages of the patients ranged from 21 to 83 years, with a median age of 48 years. The cohort included 34 males and 35 females, with six cases (8.7%) located in the spinal canal. The tumor sizes varied between 1.6 cm and 9.7 cm in maximum diameter, with an average maximum diameter of 5 cm.

### Patient cohort

Of the 69 patients, 64 had surgical records; of these, 57 underwent complete tumor resection. Additionally, 30 patients received postoperative radiotherapy, 38 did not receive postoperative radiotherapy, and one patient’s postoperative status was unknown.

### Prognosis

The follow-up period ranged from 3.2 months to 155.7 months. During this time, eight patients (11.6%) died, with a median time to death of 36.1 months (range, 15.3–126.2 months). All deceased patients had tumors classified as grade 2 or grade 3, with five patients having grade 3. A total of 15 cases (21.7%) experienced recurrence, with a median recurrence time of 36 months(range, 2.6–103 months), and the recurrence rate increased with tumor grade. Additionally, three cases (4.3%) of metastases were reported: one case of liver metastasis, one of iliac bone metastasis, and one of lung metastasis (**Table 1**).

**Table 1:** Clinicopathological features of central nervous system solitary fibrous tumors.

Variable	Grade 1	Grade 2	Grade 3
**Total cases (n=69)**	34	16	19
Sex			
Male	17	7	10
Female	17	9	9
Location			
Intracranial	31	13	19
Intraspinal canal	3	3	0
Tumor size (n=67, cm)			
<5	13	11	8
≥5	21	4	10
All cut (n=64)			
Yes	27	15	15
No	4	1	2
Radiotherapy (n=68)			
Yes	12	8	10
No	21	8	9
Follow-up			
Recurrence	5/34	3/16	7/19
Metastasis	1/34	0/16	2/19
Mortality	0/34	3/16	5/19

### Results of collagen content in CNS SFTs and its relationship with clinical pathological features and prognosis

CNS SFTs predominantly exhibited two distinct histological phenotypes: the classical SFT phenotype and the HPC phenotype. Some cases demonstrated intermediate or mixed morphology, incorporating both SFT and HPC features. Among the 69 cases, 14 displayed the classical SFT phenotype, 35 showed the HPC phenotype, and 20 exhibited a mixed phenotype. A minority of cases may exhibited rare histological patterns such as papillary and myxoid forms (28). SFTs have the potential for brain parenchymal invasion, a phenomenon observed in 19 cases in our study, with an increased incidence rate as the grade of SFT increases. Specifically, there were seven cases (20.6%) in grade 1, four cases (25%) in grade 2, and eight cases (42.1%) in grade 3.

**Figure 3:**
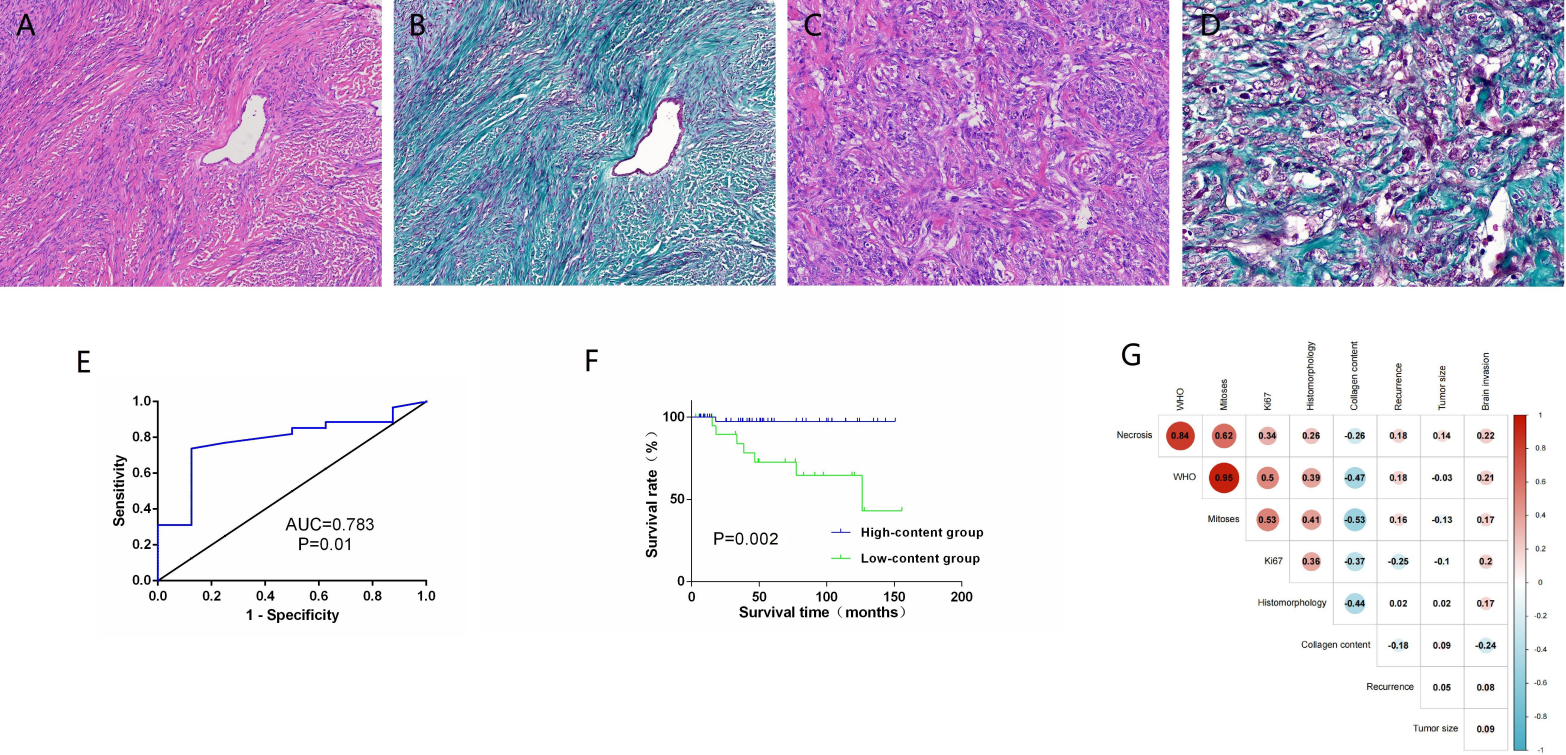
Masson’s trichrome staining highlights the collagen fibers in green. The fibers are arranged in bundles **(A, B)** or are disorganized, vary in thickness, and are wrapped around two or three tumor cells **(C, D)**. Receiver operating characteristic curve analysis reveals CNS SFTs collagen content **(E)**. Univariate survival analysis reveals a significant positive correlation between collagen content and prognosis **(F)**. Correlation heat map shows collagen content in CNS SFTs with the clinicopathological features **(G)**.

Masson’s trichrome staining revealed green (or cyan) collagen fibers, with most tumors displaying relatively uniform collagen staining. In a few cases, the intensity of collagen staining varied, particularly in tumors with mucinous changes, where collagen distribution appeared uneven. When collagen was prominent between tumor cells, it appeared in bundled, parallel structures of varying thicknesses and shapes (**Figures 3A, B**). In some cases, collagen formed scar-like nodules or radiating asbestos-like structures, while in others, it was fine and arranged around single or small clusters of tumor cells (**Figures 3C, D**). The distribution and arrangement of collagen were associated with the histological phenotype of the SFTs.

Quantitative analysis of collagen content in CNS SFTs ranged from 0.42% to 76.6% of the stained areas, with a median value of 10.72%, as calculated using IPP software. The results of HSV color model analysis showed that the stained areas proportion in the 69 cases ranged from 0% to 75%, with a median area of 11.5%. A correlation analysis between the two calculation methods revealed a significant correlation (r=0.914, *P* < 0.001). Therefore, subsequent analysis was based on the results from the HSV color model algorithm.

We conducted ROC curve analysis of collagen content, aiming to determine the optimal cutoff value for predicting OS in CNS SFTs. By evaluating various cutoff points on the ROC curve, we applied the Youden index (sensitivity plus specificity minus 1) to identify the most optimal cutoff value, which maximizes the identification rate of true positives and true negatives while minimizing misclassification. The analysis revealed that when the cutoff value was set at 6%, the Youden index reached its peak, with sensitivity and specificity value of 0.875 and 0.738, respectively (**Figure 3E**). This cutoff value stratified the patient population into high-collagen content (46 cases) and low-collagen content (23 cases) groups, providing a clear grouping criterion for subsequent survival analysis.

The log-rank survival analysis demonstrated a significant positive correlation between collagen content and OS (*P* = 0.002) (**Figure 3F**). Furthermore, the WHO grade, mitotic count, and Ki-67 demonstrated significant associations with OS, while other indicators, including histological phenotype, brain tissue invasion, and necrosis, did not achieve statistical significance in their correlation with OS (**Supplementary Table 2**). We conducted a multivariate Cox regression analysis on the seven indicators with *P* < 0.1, as identified through the log-rank test. These indicators included histological phenotype, WHO grade, brain invasion, mitotic count, necrosis, Ki-67, and collagen content. The analysis indicated that collagen content is an independent prognostic factor (*P* = 0.016), with a hazard ratio (HR) of 0.076(95% confidence interval [CI]0.009-0.617) (**Supplementary Table 2**). Based on these results, patients in the low-collagen content group had a higher risk of death than those in the high-collagen content group. Due to incomplete data regarding, recurrence, metastasis, and their respective timelines, a detailed analysis of recurrence and metastasis could not be performed.

Correlation analysis indicated that collagen content was negatively correlated with the histological phenotype, Ki67, WHO grade, mitosis, necrosis, and brain invasion (*P* < 0.05) (**Figure 3G**). The A chi-squared test revealed a significant association between collagen content and histological phenotype, Ki67, WHO grade, mitosis, and necrosis (*P* < 0.05), but no significant relationship was found with tumor size, recurrence, or brain invasion (*P* > 0.05) (**Table 2**).

**Table 2:** Relationship between collagen content and clinicopathological features.

Characteristic		Collagen content		*P*
		High	Low	
**Histological phenotype**	SFT	14	0	0.001
	HPC	17	18	
	SFT+HPC	15	5	
**WHO**	Grade 1	30	4	0.001
	Grade 2	7	9	
	Grade 3	9	10	
**Ki67**	>10%	8	13	0.002
	≤10%	38	10	
**Tumor size(cm)**	≥5	25	10	0.603
	<5	20	12	
**Mitoses:/mm^2^ (/10 HPF)**	≥5	16	19	0.000
	<5	30	4	
**Necrosis**	Yes	9	10	0.048
	No	37	13	
**Brain invasion**	Yes	10	9	0.158
	No	36	14	
**Recurrence**	Yes	8	6	0.527
	No	38	17	

WHO, World Health Organization.

### Comparison of quantitative parameters and radiomic features between high and low collagen content groups

**Figure 4:**
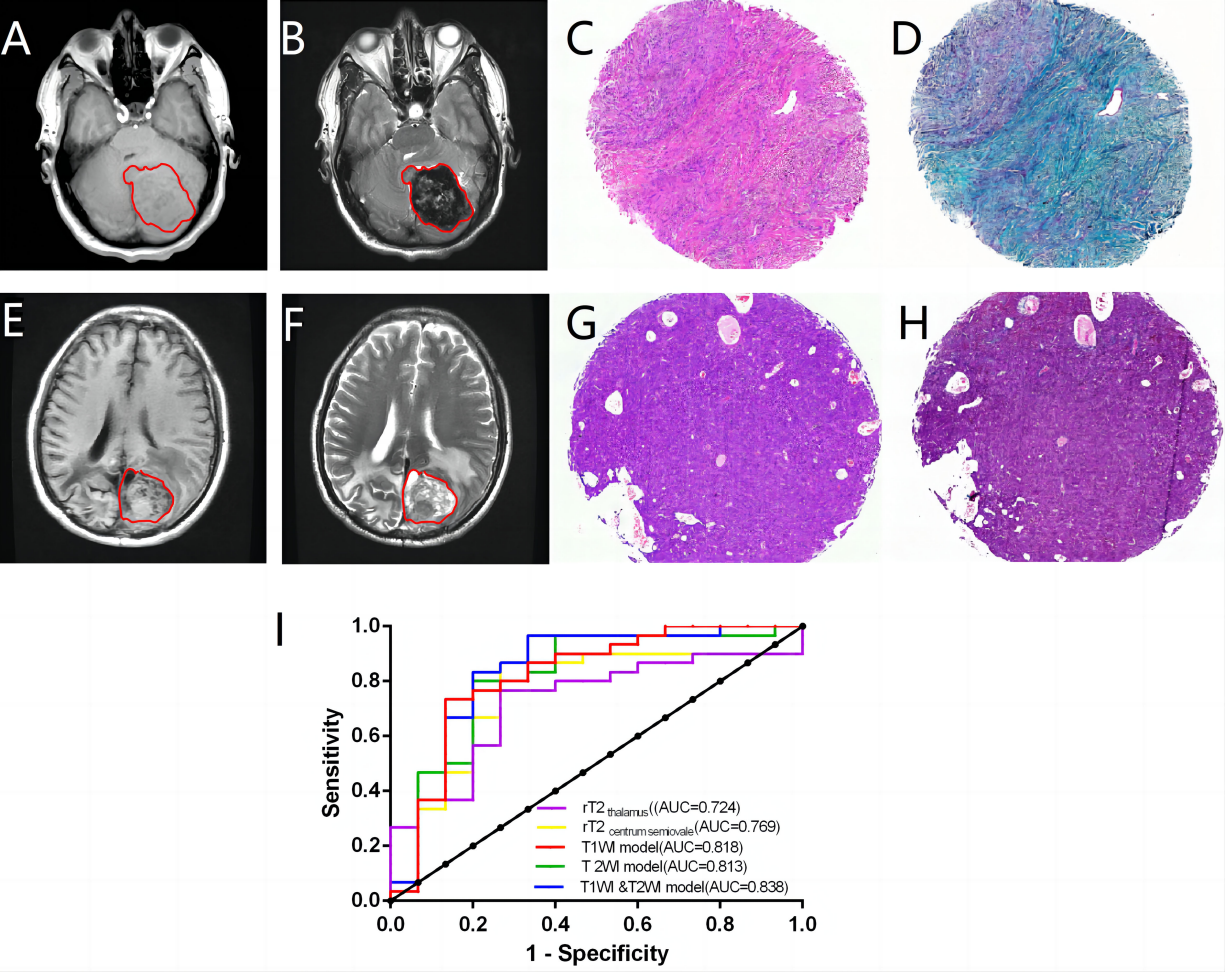
In a case with abundant collagen, the lesions display isointensity on T1WI **(A)** and heterogeneity on T2WI **(B)**. The case demonstrates a prototypical SFT phenotype characterized by abundant collagen and eosinophilic infiltration **(C)**. The collagen exhibited a green hue, following Masson’s trichrome staining, constituting approximately 60% of the tissue section **(D)**. A case with minimal collagen showing an unevenly isointense/slightly hypointense lesion on T1WI **(E)** and an unevenly slightly hyperintense lesion on T2WI **(F)**. This case has a typical “hemangiopericytoma” phenotype with minimal collagen **(G)**. The collagen content was approximately 3% **(H)**. The diagnostic efficacy of radiomics for determining collagen content is analyzed **(I)**.

In CNS SFTs, T1WI frequently displays isointensity similar to gray matter signals (**Figures 4A, E**), whereas T2WI typically exhibits signal heterogeneity (**Figures 4B, F**). The T2WI signal decreased with an increase in collagen (**Figures 4B–D**), with the tumor cell-rich areas displaying slightly higher signal values (**Figures 4F–H**). In the quantitative analysis, consistency tests demonstrated good agreement between the two observers for rT1_thalamus_, rT1_centrum semiovale_, rT2_thalamus_, and rT2_centrum semiovale_ (ICC, 0.902–0.947). Data from the more experienced observer were used for subsequent analysis. No significant differences were found in rT1 _thalamus_ and rT1_centrum semiovale_ between the high and low collagen groups (*P* > 0.05); however, significant differences were observed in rT2_thalamus_ and rT2_centrum semiovale_ between the groups (*P* < 0.05). ROC curve analysis provided diagnostic efficacy and cutoff values for rT2 _thalamus_ and rT2_centrum semiovale_ (rT2_thalamus_: AUC = 0.724 [0.571–0.847], cutoff value = 1.727; rT2_centrum semiovale_: AUC = 0.769 [0.619–0.881], cutoff value = 1.574).

In the radiomics analysis, 12 of the 1,702 radiomic features extracted for each case exhibited significant differences between the high and low collagen content groups (*P* < 0.05) (**Supplementary Table 3**). Logistic regression was used to construct T1WI, T2WI, and combined T1WI and T2WI models. The T1WI model included the T1_original_shape_Elongation and T1_wavelet-LHH_glcm_Correlation features, while the T2WI model included the T2_original_firstorder_Minimum and T2_original_shape_Elongation feature. The combined T1WI and T2WI model incorporated the T2_original_firstorder_Minimumfeature, T1_original_shape_Elongation and T1_wavelet-HLH_glcm_Idmn features. All three radiomics models demonstrated good diagnostic efficacy (AUC=0.813–0.838) (**Figure 4I**), outperforming the quantitative parameters. Although the Delong test indicated no significant differences among the models (*P*>0.05) (**Supplementary Table 4**), the combined T1WI and T2WI model exhibited the highest diagnostic efficacy, with an AUC value of 0.838, a sensitivity of 0.833, a specificity of 0.8, a PPV of 0.893, and a PPV of 0.706.

## Discussion

In recent years, research has primarily focused on CNS SFTs in relation to molecular markers, risk stratification, and the application of artificial intelligence techniques to differentiate between meningiomas and SFTs. Despite advancements in, auxiliary technologies, histological morphology remains the cornerstone of pathological diagnosis, particularly in hospitals that lack molecular detection capabilities.

Histologically, SFTs consist of varying proportions of tumor cells, collagen, and blood vessels. Collagen content is closely related to tumor cell density. Historically, the WHO grading system for SFTs was based on histological morphology. Grade 1 tumors were characterized by low tumor cell density and abundant collagen, representing the classic SFT phenotype. Grade 2 tumors exhibited higher cell density and thin-walled, “stag-horn” vessels, reflecting the classic HPC phenotype with a mitotic count of fewer than 5 mitoses per 10 high-power fields (HPF). Grade 3 tumors, representing malignant SFTs or HPCs, were defined by increased mitotic activity (≥5/10 HPF). However, the latest WHO classification of CNS SFTs has moved away from histological phenotypes, simplifying the classification by focusing on mitosis and necrosis indices.

Our study revealed that collagen content was closely correlated with the histological phenotype and the revised WHO classification of SFTs. The new classification system, which simplifies tumor grading by using mitosis and necrosis, emphasizes the prognostic importance of these markers. Mitosis, an indicator of tumor classification and prognosis (29, 30), is closely linked to tumor cell proliferation activity and cell density. Tumor necrosis is often associated with the rapid growth and proliferation of tumor cells. In our findings, we observed a negative correlation between collagen content and both mitosis and necrosis, suggesting that high collagen content serves as a favorable prognostic marker for patients with SFTs.

In addition to mitosis and necrosis, the Ki67 proliferation index is a crucial indicator of the biological behavior of tumors. Ki67 is a highly sensitive marker that more accurately and comprehensively reflects cell proliferation activity and is often negatively correlated with prognosis. In our study, Ki67 immunohistochemical staining results ranged from 1% to 40%, and we categorized them into high and low expression groups using a cutoff value of 10%. The results indicated that the Ki67 proliferation index was correlated with OS, with higher Ki67 expression associated with poorer prognosis. Both Ki67 and mitosis serve as markers of tumor proliferation activity. In our study, Ki67 and mitosis were negatively correlated with SFT collagen content.

CNS SFTs and meningiomas share similarities in their clinical presentations, imaging, and histology, and both are closely related to the meninges. The WHO classification of CNS tumors includes brain invasion as a diagnostic criterion for atypical meningiomas (31, 32). Meningioma brain invasion is characterized by irregular, tongue-like protrusions of tumor cells infiltrating the underlying parenchyma without an intervening layer of the leptomeninges. Similarly, SFTs are closely related to the meninges, and brain invasion indicates an SFTs upgrade. Based on the criteria for meningioma infiltration into the brain parenchyma, 19 cases of SFTs in our study demonstrated brain invasion, with a higher incidence in grade 3 tumors (42.1%). Our data showed that brain invasion was correlated with tumor grade, although no significant relationship was observed between brain invasion and OS or recurrence time (*P* > 0.05). Correlation analysis also revealed a negative correlation between collagen content and the incidence of brain invasion, suggesting that CNS SFTs with lower collagen content exhibit greater invasiveness.

Mitosis, necrosis, Ki67 index, and brain invasion were all related to the proliferative activity and invasiveness of SFTs and were also prognostic indicators of SFTs. Our study demonstrated a correlation between collagen content and these prognostic indicators, suggesting that collagen content serve as a reliable prognostic marker for SFTs. Histopathologically deriving the content of SFT collagen stands as the most reliable method for predicting patient prognosis in clinical practice. If non-invasive technical means to evaluate the collagen content of SFTs before surgery can be developed, predicting histological grades before surgery, formulating operation plans, and evaluating the prognosis of patients will be easier.

MRI is the most common high-resolution examination technique for brain tumors and offers a noninvasive means of evaluating tumor microenvironments preoperatively. Previous studies have demonstrated that collagen is closely associated with T2WI signal intensity. In SFTs, collagen-rich areas appear as low signal regions on T2WI, while cell-dense areas exhibit high signal intensity, a pattern often referred to as “yin and yang” or “black and white” (33). Our quantitative analysis explored the relationships between SFT signal values and collagen content on T1WI and T2WI. The results revealed that the signal values of the SFT on T2WI decreased with an increase in collagen. The AUC values of rT2 _thalamus_ and rT2_centrum semiovale_ ranged from 0.724 to 0.769, indicating moderate diagnostic efficacy. Therefore, the T2WI signal value holds promise as a predictive tool for assessing SFT collagen content.

Currently, radiomics transforms computed tomography and MRI images into exploitable high-throughput data, providing a quantitative method for describing tumor characteristics (34). Radiomics has demonstrated remarkable performance in tumor diagnosis, staging, prognosis, and treatment response prediction of tumors and in understanding the heterogeneity of tumors. In our study, radiomic methods were used to select 12 imaging features with the highest discriminatory value, primarily involving first-order and texture features after filtering transformations. The former reflects the global pixel distribution and evaluates the overall information, while the latter analyzes the spatial distribution of pixels and evaluates the local information more accurately. Therefore, combining these two can comprehensively evaluate the heterogeneity of SFTs in different dimensions (35).

In this study, radiomic data indicated higher tumor heterogeneity in the SFT group with low collagen content. We speculate that this trend may be due to the higher cell density in this group, which predominantly exhibited the HPC phenotype. These tumors are more prone to necrosis, cystic degeneration, or hemorrhage, despite efforts to avoid these regions during VOI delineation. Pathological analysis also revealed significant differences in cystic degeneration, necrosis, and hemorrhage between the high and low collagen content groups, confirming the heterogeneity between the two groups at the pathological level. In addition, differences in tumor shape, such as elongation between T1WI and T2WI sequences, suggest that tumor shape may serve as a reference value in determining collagen content. Tumor shape in the group with low collagen content group tended to have more irregular shapes, potentially reflecting faster tumor growth and greater aggressiveness. Another key finding of this study was that the diagnostic efficacy of radiomic models based on the T1WI, T2WI, and combined T1WI/T2WI models was better than that of conventional MRI quantitative parameters. The combination of the T1WI and T2WI models had the highest diagnostic efficacy, suggesting that the radiomic model using both T1WI and T2WI has the advantage of integrating information on tumor shape, and heterogeneity. It may offer a non-invasive approach to predicting collagen content in SFTs, as well as a preliminary assessment of histological phenotype and grade before surgery.

In conclusion, to the best of our knowledge, this study is the first to perform a quantitative analysis of collagen content in SFTs. We explored the relationship between collagen content in the interstitial space of SFTs, clinicopathological features, and its impact on prognosis based on morphology. The results indicate that collagen content in SFTs is a significant prognostic factor for patients. Moreover, our study demonstrated that radiomics offers greater advantages than conventional imaging data analysis in assessing collagen content and preoperative grading of SFTs. Preoperative MRI can predict collagen content and help determine the histological phenotype and grade of SFTs.

This study has several limitations. First, as a single-center retrospective study, it is subject to selection biases that may influence the interpretation of our findings. The retrospective design could have introduced biases that limit the generalizability of our results. Second, the limited number of tissue samples selected for the tissue chip may have affected the statistical power and robustness of our analyses, underscoring the need for larger sample sizes in future studies to enhance the reliability of our findings. Additionally, radiomics is sensitive to variability in imaging protocols, which may introduce confounding factors. The variability in imaging techniques across different centers could impact the consistency of our results. Furthermore, our data collection regarding recurrence and metastasis was not adequately comprehensive, leading to an incomplete analysis of the relationship between collagen content and recurrence/metastasis. This area warrants further investigation with supplementary data collection in future studies. Finally, the relatively small sample size for image analysis, may have affected the accuracy of the results. Moreover, we only analyzed data from T1WI and T2WI sequences, and we plan to include additional sequences in future studies to validate our findings.

Further research is warranted to explore potential differences in the fine structure of collagen among various grades of SFTs. To address the limitations identified in our study, we propose the following directions for future research. First, prospective trials with larger and more diverse cohorts should be conducted to validate our findings and enhance the generalizability of the results. These trials would help mitigate the effects of selection bias and provide a more comprehensive understanding of collagen’s role in SFTs. Second, multi-institutional collaborations should be pursued to increase sample size and diversity, ensuring that our findings are representative of a broader patient population. Additionally, standardization of imaging techniques across centers is crucial to reduce variability and improve the reliability of radiomic analyses. Future studies should incorporate a variety of imaging sequences, including diffusion-weighted imaging and dynamic contrast-enhanced, to provide a more comprehensive assessment of collagen’s role in tumor behavior.

Furthermore, advanced microscopy techniques, such as multiphoton microscopy, should be considered in future studies to examine the fine structure of collagen in greater detail. This approach could yield additional insights into the relationship between collagen organization and tumor aggressiveness. Lastly, more comprehensive data collection, including detailed clinical and pathological information, is needed to fully elucidate the complex interactions between collagen content and tumor behavior.

## Data Availability

The original contributions presented in the study are included in the article/**Supplementary Material**. Further inquiries can be directed to the corresponding authors.
