# Supplementary material for: Prognostic significance of collagen content in solitary fibrous tumors of the central nervous system
Source: Front Oncol. 2024 Nov 12;14:1450813. doi: 10.3389/fonc.2024.1450813 (PMC11588704; doi:10.3389/fonc.2024.1450813)
Supplement: Supplementary file 1 [file Table1.docx]

**Table S1.** Radiomics features extracted in this study.

| **Feature category** | **Feature name** |
| --- | --- |
| First-order statistics features (n = 18) | 10 Percentile, 90 Percentile, Energy, Entropy, Interquartile Range, Kurtosis, Maximum, Mean, Mean Absolute Deviation, Median, Minimum, Range, Robust Mean Absolute Deviation, Root Mean Squared, Skewness, Total Energy, Uniformity, Uniformity |
| Shape-based features (n = 14) | Elongation, Flatness, Least Axis Length, Major Axis Length, Maximum 2D Diameter Column, Maximum 2D Diameter Row, Maximum 2D Diameter Slice, Maximum 3D Diameter, Mesh Volume, Minor Axis Length, Sphericity, Surface Area, Surface Volume Ratio, Voxel Volume |
| Gray-level co-occurrence matrices (GLCM) features (n = 24) | Autocorrelation, Cluster Prominence, Cluster Shade, Cluster Tendency, Contrast, Correlation, Difference Average, Difference Entropy, Difference Variance, Id, Idm, Idmn, Idn, Imc1, Imc2, Inverse Variance, Joint Average, Joint Energy, Joint Entropy, MCC, Maximum Probability, Sum Average, Sum Entropy, Sum Squares |
| Gray-level run-length matrices (GLRLM) features (n = 16) | Gray Level Non-Uniformity, Gray Level Non-Uniformity Normalized, Gray Level Variance, High Gray Level Run Emphasis, Long Run Emphasis, Long Run High Gray Level Emphasis, Long Run Low Gray Level Emphasis, Low Gray Level Run Emphasis, Run Entropy, Run Length Non-Uniformity, Run Length Non-Uniformity Normalized, Run Percentage, Run Variance, Short Run Emphasis, Short Run High Gray Level Emphasis, Short Run Low Gray Level Emphasis |
| Gray-level size zone matrices (GLSZM) features (n = 16) | Gray Level Non-Uniformity, Gray Level Non-Uniformity Normalized, Gray Level Variance, High Gray Level Zone Emphasis, Large Area Emphasis, Large Area High Gray Level Emphasis, Large Area Low Gray Level Emphasis, Low Gray Level Zone Emphasis, Size Zone Non-Uniformity, Size Zone Non-Uniformity Normalized, Small Area Emphasis, Small Area High Gray Level Emphasis, Small Area Low Gray Level Emphasis, Zone Entropy, Zone Percentage, Zone Variance |
| Gray-level dependence matrices (GLDM) features (n = 14) | Dependence Entropy, Dependence Non-Uniformity, Dependence Non-Uniformity Normalized, Dependence Variance, Gray Level Non-Uniformity, Gray Level Variance, High Gray Level Emphasis, Large Dependence Emphasis, Large Dependence High Gray Level Emphasis, Large Dependence Low Gray Level Emphasis, Low Gray Level Emphasis, Small Dependence Emphasis, Small Dependence High Gray Level Emphasis, Small Dependence Low Gray Level Emphasis |
| Neighborhood gray-tone difference matrices (NGTDM) features (n = 5) | Busyness, Coarseness, Complexity, Contrast, Strength |

Note: 2D = two dimension; Id = inverse difference; Idm = inverse difference moment; Idmn = inverse difference moment normalized; Idn = inverse difference normalized; Imc = informal measure of correlation; MCC = maximal correlation coefficient.

**Table S2.** Univariate and multivariate analysis of prognostic factors for OS in CNS SFTs

| **Variable** | **log-rank Test** | | **Multivariate analysis** | |
| --- | --- | --- | --- | --- |
|  | **Chi-square** | ***P*** | **HR**(95%CI) | ***P*** |
| Sex | 2.069 | 0.150 | **-** | **-** |
| Age (48 years) | 0.028 | 0.866 | **-** | **-** |
| Site | 0.068 | 0.795 | **-** | **-** |
| Tumor size | 0.333 | 0.564 | **-** | **-** |
| Histological phenotype | 5.298 | 0.071 | **-** | 0.430 |
| Brain invasion | 3.496 | 0.062 | **-** | 0.129 |
| WHO grade | 7.249 | 0.027 | **-** | 0.141 |
| Collagen content | 9.800 | 0.002 | 0.076(0.009-0.617) | 0.016 |
| Ki67(10%) | 4.181 | 0.041 | - | 0.429 |
| mitotic count | 7.195 | 0.007 | - | 0.108 |
| Necrosis | 3.686 | 0.055 | - | 0.333 |

**TABLE S3.** Comparison of radiomics between SFT collagen.

| **Variables** | **Collagen content** | | ***P*** |
| --- | --- | --- | --- |
|  | **High** | **Low** |  |
| Number of cases | 31 | 16 |  |
| T1 original shape Elongation | 0.750 ± 0.103 | 0.829 ± 0.083 | 0.008 |
| T1 wavelet HLH glcmIdmn | 0.665 ± 0.089 | 0.639 ± 0.117 | 0.033 |
| T1 wavelet HLH glcmIdn | 0.988 ± 0.008 | 0.983 ± 0.009 | 0.029 |
| T1 wavelet HLH ngtdm Contrast | 0.010 ± 0.007 | 0.015 ± 0.008 | 0.018 |
| T1 wavelet LHH glcm Correlation | ﹣ 0.022 ± 0.018 | ﹣ 0.035 ± 0.018 | 0.016 |
| T1 wavelet LHL glcm Idmn | 0.991 ± 0.006 | 0.988 ± 0.004 | 0.048 |
| T1 wavelet LHL ngtdm Contrast | 0.009 ± 0.009 | 0.012 ± 0.005 | 0.033 |
| T2 original first order 10 Percentile | 355.349 ± 211.208 | 467.374 ± 159.862 | 0.043 |
| T2 original first order Minimum | 38.291 ± 75.703 | 98.622 ± 96.742 | 0.014 |
| T2 original shape Elongation | 0.750 ± 0.103 | 0.829 ± 0.083 | 0.008 |
| T2 wavelet HLL first order Mean | 0.587 ± 2.633 | ﹣ 0.404 ± 2.032 | 0.043 |
| T2 wavelet HLL first order Median | 0.445 ± 2.357 | ﹣ 0.100 ± 1.411 | 0.029 |
| T2_wavelet-LHH_glcm_Correlation | ﹣ 0.034 ± 0.019 | ﹣ 0.045 ± 0.014 | 0.026 |
| T2 wavelet LLL firstorder 10 Percentile | 1046.759 ± 607.598 | 1360.410 ± 463.861 | 0.043 |
| T2 wavelet LLL first order Minimum | 348.050 ± 282.687 | 536.999 ± 335.404 | 0.048 |

**TABLE S4.** Comparison between radiomics models.

| **Models** | **Delong Test** |
| --- | --- |
| T1&T2WI model vs rT2_thalamic_ | 0.326 |
| T1&T2WI model vs rT2 _centrum semiovale_ | 0.539 |
| T1&T2WI model vs T1WI model | 0.617 |
| T1&T2WI model vs T2WI model | 0.545 |
| T2WI model vs T1WI model | 0.929 |
| T2WI model vs rT2_thalamic_ | 0.394 |
| T2WI model vs rT2 _centrum semiovale_ | 0.671 |
| T1WI model vs rT2_thalamic_ | 0.434 |
| T1WI model vs rT2 _centrum semiovale_ | 0.674 |
| rT2 _centrum semiovale_ vs rT2_thalamic_ | 0.206 |
