# Supplementary material for: Prognostic significance of collagen content in solitary fibrous tumors of the central nervous system
Source: Front Oncol. 2024 Nov 12;14:1450813. doi: 10.3389/fonc.2024.1450813 (PMC11588704; doi:10.3389/fonc.2024.1450813)
Supplement: Supplementary file 2 [file Table2.docx]

**Table S2.** Univariate and multivariate analysis of prognostic factors for overall survival in solitary fibrous tumors.

| **Variable** | **log-rank Test** | | **Multivariate analysis** | |
| --- | --- | --- | --- | --- |
|  | **Chi-square** | ***P*** | **HR**(95%CI) | ***P*** |
| Sex | 2.069 | 0.150 | **-** | **-** |
| Age (48 years) | 0.028 | 0.866 | **-** | **-** |
| Site | 0.068 | 0.795 | **-** | **-** |
| Tumor size | 0.333 | 0.564 | **-** | **-** |
| Histological phenotype | 5.298 | 0.071 | **-** | 0.430 |
| Brain invasion | 3.496 | 0.062 | **-** | 0.129 |
| WHO grade | 7.249 | 0.027 | **-** | 0.141 |
| Collagen content | 9.800 | 0.002 | 0.076(0.009-0.617) | 0.016 |
| Ki67(10%) | 4.181 | 0.041 | - | 0.429 |
| mitotic count | 7.195 | 0.007 | - | 0.108 |
| Necrosis | 3.686 | 0.055 | - | 0.333 |
