# Supplementary material for: Prognostic significance of collagen content in solitary fibrous tumors of the central nervous system
Source: Front Oncol. 2024 Nov 12;14:1450813. doi: 10.3389/fonc.2024.1450813 (PMC11588704; doi:10.3389/fonc.2024.1450813)
Supplement: Supplementary file 3 [file Table3.docx]

**TABLE S3.** The radiomics features of solitary fibrous tumors between high and low collagen content group.

| **Variables** | **Collagen content** | | ***P*** |
| --- | --- | --- | --- |
|  | **High** | **Low** |  |
| Number of cases | 30 | 15 |  |
| T1_original_shape_Elongation | 0.752 ± 0.104 | 0.831 ± 0.085 | 0.011 |
| T1_wavelet-HLH_glcm_Idmn | 0.989 ± 0.007 | 0.984 ± 0.008 | 0.034 |
| T1_wavelet-HLH_glcm_Idn | 0.938 ± 0.020 | 0.924 ± 0.021 | 0.030 |
| T1_wavelet-HLH_ngtdm_Contrast | 0.010 ± 0.007 | 0.015 ± 0.008 | 0.018 |
| T1_wavelet-LHH_glcm_Correlation | ﹣0.022 ± 0.019 | ﹣0.035 ± 0.018 | 0.032 |
| T1_wavelet-LHL_glcm_Idmn | 0.991 ± 0.006 | 0.988 ± 0.004 | 0.034 |
| T1_wavelet-LHL_glcm_Idn | 0.945 ± 0.017 | 0.935 ± 0.013 | 0.032 |
| T1_wavelet-LHL_ngtdm_Contrast | 0.009 ± 0.009 | 0.012 ± 0.005 | 0.020 |
| T2_original_first order_Minimum | 31.967 ± 68.163 | 99.863 ± 100.005 | 0.010 |
| T2_original_shape_Elongation | 0.752 ± 0.104 | 0.831 ± 0.086 | 0.011 |
| T2_wavelet-HLL_firstorder_Median | 0.407 ± 2.388 | ﹣0.100 ± 1.460 | 0.043 |
| T2_wavelet-LLL_first order_Minimum | 337.116 ± 280.773 | 549.039 ± 343.579 | 0.041 |

Note: glcm = gray-level co-occurrence matrices; Idmn = inverse difference moment normalized; Idn = inverse difference normalized; ngtdm = neighborhood gray-tone difference matrices.
