# Supplementary material for: Prognostic significance of collagen content in solitary fibrous tumors of the central nervous system
Source: Front Oncol. 2024 Nov 12;14:1450813. doi: 10.3389/fonc.2024.1450813 (PMC11588704; doi:10.3389/fonc.2024.1450813)
Supplement: Supplementary file 4 [file Table4.docx]

**TABLE S4.** Comparison of diagnostic performance among logistic regression models in predicting collagen content in solitary fibrous tumors.

| **Models** | **Delong Test** |
| --- | --- |
| T1WI&T2WI model vs rT2_thalamic_ | 0.326 |
| T1WI &T2WI model vs rT2 _centrum semiovale_ | 0.539 |
| T1WI &T2WI model vs T1WI model | 0.617 |
| T1WI &T2WI model vs T2WI model | 0.545 |
| T2WI model vs T1WI model | 0.929 |
| T2WI model vs rT2_thalamic_ | 0.394 |
| T2WI model vs rT2 _centrum semiovale_ | 0.671 |
| T1WI model vs rT2_thalamic_ | 0.434 |
| T1WI model vs rT2 _centrum semiovale_ | 0.674 |
| rT2 _centrum semiovale_ vs rT2_thalamic_ | 0.206 |

Note: T1WI = T1-weighted imaging; T2WI = T2-weighted imaging; rT2_thalamic_ = T2 value of the tumor/T2 value of the thalamus; rT2 _centrum semiovale_ = T2 value of the tumor/T2 value of the centrum semiovale.
